# Recruitment and participant baseline characteristics in the dialysis outcomes in those aged 65 years or older study

**DOI:** 10.1186/s12882-019-1328-8

**Published:** 2019-04-23

**Authors:** Bronwen McNoe, John B. W. Schollum, Sarah Derrett, Mark R. Marshall, Andrew Henderson, Ari Samaranayaka, Robert J. Walker

**Affiliations:** 1 Department of Preventive and Social Medicine, University of Otago, PO Box 56, Dunedin, 9054 New Zealand; 2 Department of Medicine, University of Otago, Dunedin, New Zealand; 3 Department of Renal Medicine, Middlemore Hospital, Counties Manukau District Health Board, Manukau, New Zealand; 4 Renal Medicine, Hamilton Hospital, Waikato District Health Board, Hamilton, New Zealand; 5 Biostatistics unit, University of Otago, Dunedin, New Zealand

**Keywords:** Dialysis, Elderly, Quality of life, Comorbidities

## Abstract

**Background:**

Despite an increasing number of older people commencing dialysis the impact of dialysis on their quality of life and survival, remains unclear. The Dialysis Outcomes in those aged over 65 years or older study is an accelerated prospective cohort longitudinal design study, designed to obtain sufficient health related quality of life data, linked to clinical data, to inform clinicians’ and patients’ decision-making with respect to end stage kidney disease (ESKD), outcomes, and options for management in New Zealand (NZ).

**Methods:**

The study has an accelerated prospective cohort longitudinal design, comprised of cross-sectional and longitudinal components. We report the baseline data on the 225 participants enrolled in the study. Dialysis duration was grouped in tertiles from less than one year (incident patients), 1–3 years and greater than 3 years. Health related quality of life data was obtained from self-reported questionnaires including KDQoL-36, EQ-5D-3 L, FACIT, WHODAS II, and the Personal Well-being Score.

**Results:**

The median age of the cohort was 71 years and two thirds were male. Three quarters of the participants were on dialysis at the baseline, with 42% of those on home dialysis (haemodialysis or peritoneal dialysis). Māori and Pacific people were over represented (20% Māori and 24% Pacific) in the sample, when compared to the general NZ population of the same age group (where 5% are Māori and 2% are Pacific). At baseline, there were no differences observed in sociodemographic, quality of life or health characteristics between the dialysis groups either by modality or duration of dialysis.

**Conclusions:**

We report the baseline characteristics of participants enrolled prospectively into a longitudinal cohort observational study examining health related quality of life factors with clinical characteristics on dialysis outcomes in a group of New Zealanders aged 65 years or older who are either on dialysis or have been educated about dialysis (BMC Nephrol 14:175, 2013). Subsequent publications are planned, analysing the prospective longitudinal data to identify key factors that determine both outcome and quality of life for individuals of this age group.

**Trial registration:**

ACTRN12611000024943.

## Background

Over the past two decades there has been a considerable increase in the proportion of older patients commencing dialysis; yet there is some uncertainty about the outcome of dialysis in this population with respect to survival and also patients’ health-related quality of life (HRQoL). There is currently minimal prospective data published on the key factors that might influence decision making or outcomes in the older age groups commencing renal replacement therapy. More recently, the SONG (Standardised Outcomes in NephroloGy) initiative has identified the importance of patient-reported outcome measures in studies of patients on dialysis [1]. It is therefore important to identify those factors to inform shared decision making.

The Dialysis Outcomes in those aged 65 years or older study (DOS65) is an accelerated prospective cohort longitudinal study, designed to obtain sufficient HRQoL data, linked to clinical data, to inform clinicians’ and patients’ decision-making with respect to end stage kidney disease (ESKD) outcomes and options for management in New Zealand (NZ) [2]. This study commenced in January 2010 and final follow up was completed in June 2016.

The focus of this paper is to describe the key characteristics of the study cohort at baseline, including the patients’ health status, their treatment modality (not on dialysis, haemodialysis or peritoneal dialysis) and location of treatment (i.e. home or clinic/hospital facility). From the cross-sectional data at baseline, we hypothesise that HRQoL differs between patients according to: sex, ethnicity, comorbidities, type of dialysis treatment, health service satisfaction, and duration of dialysis.

## Methods

### Study design

**Fig. 1:**
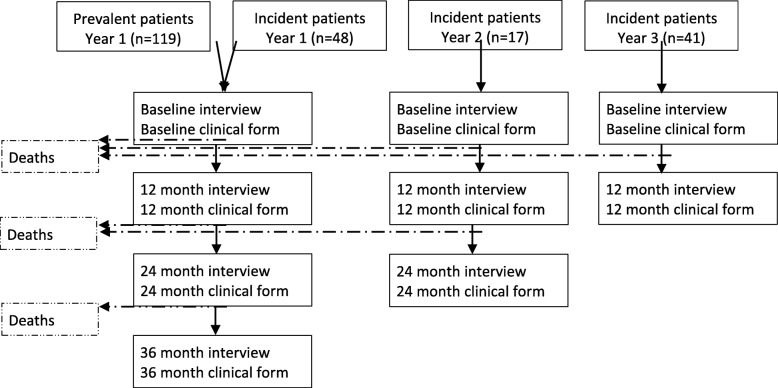
Accelerated prospective cohort longitudinal design of the study, comprised of cross-sectional and longitudinal components with baseline recruitment numbers for participants over the 3 years of the study

The study has an accelerated prospective cohort longitudinal design, comprised of cross-sectional and longitudinal components (Fig. 1). The methods have been described previously [2]. In particular, individuals who had a very limited life expectancy or significant active co-morbidity which was affecting their health were excluded by principal investigator at each site, as this would clearly impact on the perceived HRQoL questionnaires, but may not be attributable to dialysis and ESKD specifically. Recruitment was targeted to try and get at least one set of interviews at 12 months after enrolment for all participants where possible.

### Setting and participants

Eligible patients resided in one of three District Health Board (DHB) regions within NZ (Counties Manukau, Hawkes Bay or Southern) which reflect the diversity of the New Zealand population with Counties Manukau, a large urban population with high representation of Māori and Pacific people; Hawkes Bay, a regional farming centre which also has a high proportion of Māori and the Southern region with a predominantly European population spread over a large demographic area. All participants were 65 years or older, their clinical team considered them well enough to be approached to participate, and were either established on dialysis at the baseline interview or had a eGFR 15 ml/min 1.73 m^2^ or less and had commenced pre-dialysis education. In practice, this meant that some patients were already on dialysis for a number of years at the time of recruitment, whereas other patients had just commenced treatment. Clinicians from each DHB approached eligible patients and invited them to participate in the study. Ethical approval was granted by the New Zealand Health and Disability Ethics Committee (MEC/10/08/084) and the study is registered with Australian and New Zealand Clinical Trials Registry (ACTRN12611000024943).

### Data collection

Following consent, the patients were telephoned by a trained interviewer to arrange an interview either face to face or by telephone depending on the participants’ preference. Interview questions were read aloud to participants, who were also provided with a printed sheet of response options to facilitate the interviews. The baseline interview was predominantly comprised of structured closed-response questions (see below).

Clinical data was collected at the same time as the first (baseline) interview for each participant. Clinicians completed a standardised clinical data abstraction form based on report forms used for the Dialysis Outcomes and Practice Patterns Study. Data collected included ethnicity, age, sex, co-morbidities, laboratory data, anthropometric measures, prescribed medications and details of renal replacement therapy [3] In addition, we obtained aggregate data from routinely collected clinical and administrative hospital datasets included in the Australian and New Zealand Dialysis and Transplantation Registry (ANZDATA) to compare characteristics of our cohort with the broader New Zealand wide dialysis population in this age group [4].

### Variables

We collected socio-demographic characteristics using the same questions used in the 2009 New Zealand census: sex, age, relationship status, occupation, living arrangements and self-reported ethnicity [5]. As participants were able to select multiple ethnicities, ethnicity was prioritised into Māori (New Zealand’s indigenous population), Pacific peoples (Samoan, Tongan, Fijian, Cook Islander), other ethnicities (e.g. other European, Chinese, Indian) or New Zealand European [5, 6]. Socioeconomic data included home ownership [5], whether income was solely dependent on Government Superannuation [7], use of a means-tested community services card (to provide additional subsided health care) [8], self-reported standard of living (responses: high, fairly high, medium, fairly low, low) [9], adequacy of household income to meet every day needs (not enough, just enough, enough or more than enough), and education (the responses were merged into 3 categories: none, secondary (age 12–18 years), post-secondary) [9].

The New Zealand Deprivation Index (NZDep2006) is calculated for small New Zealand geographical areas using 9 dimensions of socio-economic disadvantage to create a summary score to estimate socioeconomic status using data collected in Statistics New Zealand’s 2006 Census of Population and Dwellings [10]. The higher the number (range 1–10) the greater the estimated socioeconomic deprivation.

General health status was assessed using the EQ-5D-3 L [11], as previously used in studies of Chronic Kidney Disease (CKD) populations [11, 12]. The Kidney Disease Quality of Life - 36 (KDQoL-36) was used to assess the functioning and well-being of people with CKD [12–14].

We collected responses to the Functional Assessment of Chronic Illness Therapy (FACIT) survey, which is a collection of health-related quality of life measures targeted to the management of chronic disease and has been validated in elderly populations [15]. The higher the score the higher the level of satisfaction with health care. A total FACIT score was calculated as a simple (unweighted) sum of all the subscale scores, rescaled to a 0 to 100 scale. This was calculated only for people with valid sub-scales for all dimensions.

The level of disability for participants was measured using the WHODAS II 12-item measure which assesses activity limitations and participation over the past 30 days using a five point Likert scale (“none/mild/moderate/extreme/cannot do”) for each item. [16] A WHODAS disability score was derived using the summed approach (where 0 = no disability and 48 = disability). When one WHODAS item was missing, the individual’s average was imputed; when more items were missing, the disability score was not derived. [16]

Well-being was measured using the Personal Wellbeing Index [17]. This scale is an eight-item measure assessing the level of satisfaction with; standard of living, health, achieving in life, relationships, safety, community connectedness, future security and spirituality or religion. Respondents rated their satisfaction on an 11 point scale (0 completely dissatisfied to 10 completely satisfied). Scores for individuals were derived as an average across all items and converted to a zero to 100 scale.

### Data analysis

Data analysis was conducted Stata® 13.1 software (StataCorp. 2013. *Stata Statistical Software: Release 13*. College Station, TX, USA. Descriptive statistics were calculated. ANOVA (for continuous data) and Chi Square (for categorical data) were used to compare groups (e.g. non-responders versus responders).

## Results

### Participants

**Fig. 2:**
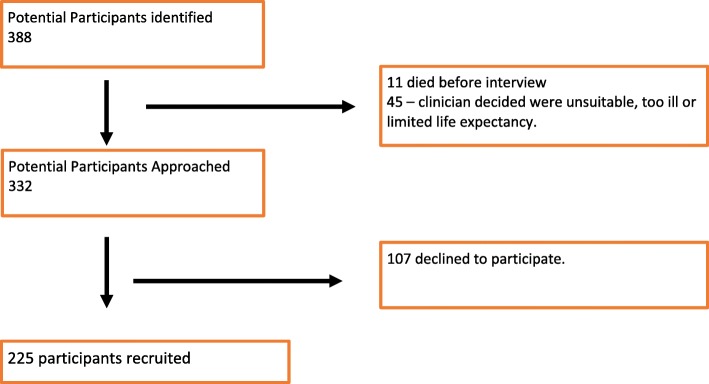
Flow-chart demonstrating recruitment for the study

Between 1 January 2010 and 31 March 2014 there were 388 potential participants of whom 56 were ineligible for the first interview because; they died before interview was scheduled (*n* = 11) or a clinician advised interview would be inappropriate given the poor health status of the patient or anticipated limited life expectancy (*n* = 45). Of the 332 potential participants, 107 (32%) declined to participate in the study, leaving 225 (68%) participants (Figs. 1 & 2). The demographic characteristics of patients who agreed and declined to participate in the study is provided in Table 1. There were a number of statistically significant differences between the two groups. Participants were more likely to be male, of European ethnicity and on dialysis. Over half of the 225 participants (54%) completed a face to face interview, 45% by telephone and 1 completed a postal questionnaire. Interviews took on average one hour to complete. 25 participants (11%) had limited or no English language ability and were assisted by a bi-lingual translator.

**Table 1:** Comparison of participants and non-participants

		Participated (*N* = 225)		Declined to participate (*N* = 107)		chi^2^ *p* value
		n	%	n	%	
Gender						
Male		144	64.0	49	45.8	
Female		81	36.0	58	54.2	0.002
Age group						
65–69		87	38.7	50	46.7	
70–74		64	28.4	36	33.6	
75–79		45	20.0	12	11.2	0.097
80+		29	12.9	9	8.4	
median age		71	–	70	–	
Ethnicity (as in ANZDATA)						
European		108	48.0	11	10.3	
Māori		45	20.0	24	22.4	
Pacific		53	23.6	52	48.6	0.000
Other		19	8.4	19	17.8	
Missing		0	0.0	1	0.9	
Ethnicity (Self reported multiple ethnicities were prioritised)						
Europeans		96	42.7	–		
Māori		50	22.2			
Pacific		52	23.1			–
Other		27	12.0			
Receiving dialysis						
Yes		169	75.1	68	63.6	
No		56	24.9	39	36.4	0.029
Living Arrangement						
Alone		33	14.7	–		–
Living with others		192	85.3			
Cigarette smoking						
Current smoker		14	6.2	–		
Ex smoker		118	52.4			
Never smoker		91	40.4			–
Missing		2	0.9			
Alcohol drinker						
Current drinker		96	42.7	–		–
Non drinker		129	57.3			
Highest educational qualification						
None		108	48.0	–		
Secondary (age 13–18)		44	19.6			
Post secondary		69	30.7			–
Missing		4	1.8			
Comorbidities^a^						
Cardiovascular disease		151	67.1	68	63.6	0.52
Cerebrovascular disease		17	7.6	4	3.7	0.18
Peripheral vascular disease		45	20.0	21	19.6	0.94
Diabetes mellitus		120	53.3	80	74.8	< 0.01
Respiratory disease		49	21.8	12	11.2	0.02
Cancer (other than skin cancer)		38	16.9	8	7.5	0.02
Musculoskeletal		68	30.2	32	29.9	0.95
Other		140	62.2	81	75.7	0.02
Comorbidity count						
0 to 2		104	46.2	40	37.4	
3 to 6		121	53.8	67	62.6	0.13
Cause of ESKD						
Glomerulonephritis		38	16.9	15	14.0	
Hypertension		38	16.9	10	9.3	
Polycystic kidney		12	5.3	0	0.0	< 0.01
Diabetes mellitus		92	40.9	68	63.6	
Other		45	20.0	14	13.1	

^a^multiple comorbidities possible

A comparison of DOS65’s population with the entire comparative age group on dialysis in NZ [4], found there was no significant difference in the distribution according to age or sex. In DOS65, 42% were dialysing at home which reflects national trends [4]. When compared to the New Zealand wide dialysis population of the same age [4], DOS65 had a higher proportion of Pacific participants (23% vs 17%), a similar proportion of Māori participants (22% vs 25%) and a slightly lower proportion of New Zealand Europeans (43% vs 49%). When compared to the general NZ population over the age of 65, Māori (22% vs 5%) and Pacific people (23% vs 2%) were over represented in the study population [9].

Participant characteristics are summarised in Table 1. Nearly two thirds of the study cohort were male and the median age at interview was 71 years (interquartile range, IQR 68 to 76 years). Three quarters (*n* = 169) of the patients were receiving dialysis (109 haemodialysis and 60 peritoneal dialysis). Of those on dialysis, 70 (41%) were dialysing at home; and 99 (59%) underwent centre-based haemodialysis. 119 (70%) had been dialysing for more than one year at baseline; 57 (34%) had been dialysing for between 1 and 3 years and 62 (37%) had been dialysing for more than 3 years. Fifty (30%) were incident dialysis patients (started dialysis within the past 12 months). Of the 56 non-dialysis patients, 9 had elected for conservative treatment (i.e. had made a conscious choice to not go onto dialysis), and 47 were defined as pre-dialysis. There was no sub-cohort effect observed across dialysis duration (Table 2). Of the pre-dialysis patients, 5 had been deemed medically unfit to start dialysis, 41 had chosen to dialyse but had not reached the point at which dialysis needed to be started, and 1 patient had not yet made up his/her mind about whether to dialyse or not.

**Table 2:** Assessing possibility of subcohort effect in accelerated prospective design

		Predialysis (*n* = 56)		Incident (< 1 year) (*n* = 50)		Prevalent (1–3 years) (*n* = 57)		Prevalent (> 3 years) (*n* = 62)		*p* value
		n	%	n	%	n	%	n	%	
Gender										
Male		37	66.1	33	66.0	35	61.4	39	62.9	
Female		19	33.9	17	34.0	22	38.6	23	37.1	0.94
Age group										
65–69		20	35.7	20	40.0	21	36.8	26	41.9	
70–74		11	19.6	16	32.0	15	26.3	22	35.5	
75–79		13	23.2	6	12.0	16	28.1	10	16.1	0.13
80+		12	21.4	8	16.0	5	8.8	4	6.5	
Ethnicity (self reported)										
European		30	53.6	19	38.0	23	40.4	24	38.7	
Māori		10	17.9	10	20.0	18	31.6	12	19.4	
Pacific		6	10.7	17	34.0	10	17.5	19	30.6	0.06
Other		10	17.9	4	8.0	6	10.5	7	11.3	
Comorbidities^a^										
Cardiovascular disease		36	64.3	33	66.0	38	66.7	44	71.0	0.88
Cerebrovascular disease		4	7.1	3	6.0	5	8.8	5	8.1	0.97
Peripheral vascular disease		13	23.2	7	14.0	11	19.3	14	22.6	0.63
Diabetes mellitus		27	48.2	29	58.0	32	56.1	32	51.6	0.74
Respiratory disease		16	28.6	10	20.0	9	15.8	14	22.6	0.43
cancer (other than skin cancer)		8	14.3	11	22.0	8	14.0	11	17.7	0.67
Musculoskeletal		18	32.1	14	28.0	14	24.6	22	35.5	0.60
Other		36	64.3	30	60.0	37	64.9	37	59.7	0.91
Comorbidity count										
0 to 2		24	42.9	23	46.0	29	50.9	28	45.2	
3 to 6		32	57.1	27	54.0	28	49.1	34	54.8	0.85
Cause of ESKD										
Glomerulonephritis		8	14.3	9	18.0	7	12.3	14	22.6	
Hypertension		10	17.9	7	14.0	12	21.1	9	14.5	
Polycystic kidney		2	3.6	1	2.0	4	7.0	5	8.1	
Diabetes mellitus		20	35.7	22	44.0	27	47.4	23	37.1	0.55
Other		16	28.6	11	22.0	7	12.3	11	17.7	

^a^multiple comorbidities possible

Diabetes mellitus was the most common cause of ESKD (41%) followed by glomerulonephritis (17%) and hypertensive disease (17%). The majority of participants had 3 or more comorbidities, of which cardiovascular disease (CVD) (67%), followed by diabetes mellitus (53%) were the most common. The number of comorbidities ranged from zero to 6 with a median of 3 (IQR 2 to 4) (Table 1). Over half the population had previously smoked (52%) though there were very few current smokers (6%). The population was less well educated than the general NZ population of the same age with 48% of the study population not having achieved a secondary school education as compared with 39% of the NZ population over the age of 65 (2013 NZ Census). The study population lived in more deprived areas than the general NZ population over the age of 65. DOS65 participants were poorer socioeconomically, according to the NZDep, than the general population (6.9 and 5.4 respectively, *p* < 0.01), and more than half (54%) lived in areas classified as having the poorest three deciles. (Personal communication Dr. June Atkinson Department of Public Health, University of Otago Wellington, NZ; data sourced from Statistics New Zealand).

Table 3 describes participants’ sociodemographic characteristics by baseline renal replacement therapy, dialysis duration and location of treatment. The length of time on dialysis (dialysis vintage) did not vary by gender, age, ethnicity, distance from dialysis facility or any makers of socioeconomic status. In contrast dialysis modality and location was highly associated with socio-demographic factors. Female participants were less likely to dialyse at home than males (27 vs 49%). When compared to the NZ European population a smaller proportion of Māori (25 vs 61%) or Pacific people (24vs 61%) dialysed at home. Participants living further away from the dialysis centre were more likely to undertake a home therapy, particularly peritoneal dialysis. Patients dialysing in-centre (compared to those dialysing at home), were more likely to be socioeconomically disadvantaged as indicated by not owning their own home (72 vs 48%), being more likely to have a community services card (85%vs 62%), reporting a low/very low standard of living (14% vs 3%) and living in a more deprived NZDep area (mean 7.6 vs 6.1). Age had no impact on dialysis location and people living alone were no less likely to undertake a home therapy. Patients dialysing at home had a similar number of comorbidities to those dialysing in-centre. The characteristics of the pre-dialysis population was similar to the population on peritoneal dialysis. Females, Māori and Pacific people, those who lived closer to a dialysis centre, and those who were socioeconomically poorer, were more likely to undertake haemodialysis when compared to the peritoneal dialysis or predialysis population.

**Table 3:** Sociodemographic characteristics of study cohort by type of treatment, location and time on treatment

	Not on dialysis (N = 56)	Type of dialysis			Dialysis Location**			Time on dialysis**			
Characteristic		Haemodialysis (*n* = 109)	Peritoneal (*n* = 60)	p value *	Home (*n* = 70)	In centre (*n* = 99)	p value *	Incident (< 1 year) (n = 50)	Prevalent (1–3 years) (n = 57)	Prevalent (> 3 years) (n = 62)	p value *
Gender											
Male	37	63	44	*0.045*	53	54	*0.005*	33	35	39	*0.883*
Female	19	46	16		17	45		17	22	23	
Age group											
65–69	20	43	24		29	38		20	21	26	
70–74	11	35	18	*0.729*	22	31	*0.808*	16	15	22	*0.262*
75–79	13	22	10		11	21		6	16	10	
≥ 80	12	9	8		8	9		8	5	4	
Prioritised ethnicity											
European	30	32	34		40	26		19	23	24	
Māori	10	31	9	*0.005*	10	30	*0.000*	10	18	12	*0.457*
Pacific	6	35	11		11	35		17	10	19	
Other	10	11	6		9	8		4	6	7	
Distance in km to dialysis centre											
Less than 10	30	65	27		29	63		28	31	33	
10–49	19	39	23	*0.019*	26	36	*0.000*	16	23	23	*0.739*
50+	7	5	10		15	0		6	3	6	
Lifestyle behaviour											
Current smoker	4	7	3	*0.708*	4	6	*1.000*	2	2	6	*0.287*
Currently drinks alcohol	30	39	27	*0.240*	38	28	*0.001*	19	22	25	*0.965*
Lives alone	16	10	7	*0.606*	7	10	*1.000*	2	6	9	*0.182*
Own home											
Yes	37	57	43	*0.040*	51	49	*0.008*	31	35	34	*0.505*
No	19	51	17		19	49		18	22	28	
Missing	0	1	0		0	1		1	0	0	
Pension is the only source of income											
Yes	35	80	40	*0.309*	45	75	*0.083*	32	44	44	*0.400*
No	21	28	20		25	23		17	13	18	
Missing	0	1	0		0	1		1	0	0	
Have a community services card?											
Yes	43	85	42	*0.505*	44	83	*0.007*	34	44	49	*0.210*
No	13	23	17		25	15		14	13	13	
Missing	0	1	1		1	1		2	0	0	
Standard of living											
Fairly Low/Low	4	15	2	*0.033*	3	14	*0.034*	5	5	7	*0.901*
High/fairly high/medium	52	93	58		67	84		44	52	55	
Missing	0	1	0		0	1		1	0	0	
Adequacy of income for daily needs											
Enough/More than enough	27	50	26	*0.712*	31	45	*0.834*	21	30	25	*0.373*
Not enough/Just enough	29	58	34		39	53		28	27	37	
Missing	0	1	0		0	1		1	0	0	
Qualifications	26	56	26	*0.217*	31	51	*0.233*	24	27	31	*0.850*
None	30	49	34		39	44		25	30	28	
Secondary school/higher/trade qualification	0	4	0		0	4		1	0	3	
Missing											
Comorbidity Count											
0–2	24	52	28	*0.900*	32	48	*0.722*	23	29	28	*0.802*
3+	32	57	32		38	51		27	28	34	
NZDep score (mean & SD)	6.6(2.9)	7.3(2.8)	6.2(3.1)	0.016	6.1(2.9)	7.6(2.8)	0.001	7.0(3.1)	6.9(2.9)	6.9(2.9)	0.98

*P values are from chi-square test except for NZDep score where *P* values are from ANOVA. Non-dialysis group now excluded from test when comparing dialysis types, ** 8 people are in ‘in training’ for dialysis. Two of them were included in “home” group as they are training at home, other 6 were included in “centre” group

Table 4 presents self-reported HR-QOL, disability and personal well-being measures at baseline. With the exception of dialysis location on EQ-5D-3 L VAS, these self-reported scales of health and disability were not impacted by being on dialysis or by dialysis modality, location or duration.

**Table 4:** Health and disability measures by type of treatment, location and time on treatment

	Not on dialysis (N = 56)	Type of dialysis			Location			Time on dialysis			
Characteristic		Haemodialysis (n = 109)	Peritoneal (n = 60)	p value *	Home (n = 70)	In centre (n = 99)	p value *	Incident (< 1 year) (n = 50)	Prevalent (1–3 years) (n = 57)	Prevalent (> 3 years) (n = 62)	p value *
EQ5D_VAS Health state today(median & IQR)	70 (50 to 80)	70 (60 to 80)	67.5 (50 to 80)	0.54	62.5 (50 to 80)	70 (60 to 80)	0.01	67.5 (50 to 80)	70 (60 to 80)	70 (50 to 80)	0.49
FACIT total score (median & IQR	91.4 (83.9 to 96.6)	90.5 (78.2 to 95.3)	90.2 (84.7 to 96.0)	0.90	90.1 (83.6 to 95.9)	91.2 (78.2 to 95.8)	0.64	85.7 (74.5 to 95.9)	90.1 (83.0 to 95.0)	91.7 (82.4 to 96.3)	0.09
Personal Wellbeing Index total score (median & IQR)	76.9 (66.3 to 87.5)	78.8 (68.8 to 87.5)	76.3 (62.5 to 83.8)	0.45	76.3 (62.5 to 83.8)	80 (68.9 to 87.5)	0.23	79.4 (65.0 to 85.6)	75.0 (65.0 to 83.8)	78.8 (70.0 to 87.5)	0.49
WHODAS score (median & IQR).	10.0 (5.0 to 15.0)	11.0 (6.0 to 17.5)	11.5 (5.0 to 19.0)	0.73	10.5 (5.0 to 19.0)	11.0 (6.0 to 18.0)	0.73	11.0 (5.0 to 17.0)	11.5 (8.0 to 16.0)	11.5 (5.0 to 19.0)	0.88
Kidney symptom score (Median & IQR)	78.4 (62.5 to 88.6)	81.3 (66.6 to 87.5)	77.1 (62.5 to 87.5)	0.15	79.2 (64.6 to 87.5)	81.3 (66.7 to 89.6)	0.53	79.2 (60.4 to 85.4)	81.3 (64.6 to 87.5)	81.3 (68.8 to 89.6)	0.87

There were no missing values for EQ5D_VAS or the Kidney symptom score, 2 participants had missing WHODAS values, 21 participants had missing FACIT values (16 in the non dialysis group) and 29 participants had missing values from the Personal Wellbeing Index

## Discussion

The DOS65 study is an accelerated prospective cohort longitudinal design study over 3 years, designed to obtain HRQoL data, which when linked to clinical data, will help inform clinicians’ and patients’ shared decision-making with respect to end stage kidney disease (ESKD), outcomes and options for management in New Zealand (NZ) [2]. The outcomes of this study align well with the SONG initiative, and the importance of reporting patient-related outcomes [1]. This paper reports the baseline characteristics of the study cohort.

Patients who participated in the study were more likely (than those who declined to participate) to be male, of NZ European descent and on dialysis treatment. However, despite this, by selecting 3 different regions with differing population characteristics, we achieved a reasonable representation of the NZ dialysis population in this age group. Given the much higher incidence and prevalence of ESKD (3.5–5.5 fold) [18] and CKD [19] among Māori and Pacific populations, and differences in outcome measures and HRQoL by ethnicity [20–23], it was essential that good representation of these populations was achieved.

While diabetes mellitus was the most common cause of ESKD this was less prevalent than in the general NZ dialysis population [4]. As one would expect, this is a highly comorbid population. When compared to the DOPPS population over 65, comorbidity rates were broadly similar [24], with more than half the study populations having cardiac disease. This population had a higher rate of diabetes mellitus but lower rates of cerebrovascular and peripheral vascular disease. The increased prevalence of diabetes mellitus reflects the participation of Māori and Pacific people with well documented increased propensity to ESKD as a result of diabetes mellitus [18].

In the general population socioeconomic deprivation plays an important role in the development and progression of kidney disease, and conversely, chronic kidney disease may result in socioeconomic deprivation [20, 25, 26]. Study participants were much more likely to live in a more deprived area, were less well educated, and were financially less well off than other New Zealanders of a similar age. While the association of socioeconomic disadvantage and renal replacement therapy is well established in the general population, Australian data has previously suggested that this is less relevant in older dialysis patients [27]. To our knowledge this is the first cohort to describe an association between socioeconomic disadvantage and renal replacement therapy in this population. The main factor influencing where participants resided and hence deprivation index was ethnicity. Māori and Pacific people were much more likely to live in the two most socioeconomically deprived deciles with 68% of Māori and 81% of Pacific participants living in decile 9 or 10 areas as opposed to 13% of NZ Europeans.

New Zealand has among the highest rates of home-based dialysis internationally, even among older age groups. [4]. In this cohort, 42% were dialysing at home which reflects the national trend [4]. Most patients dialysing at home were on PD, with only 10 participants on home HD. We found that patients dialysing at home were more likely to own their own home, more likely to have more than one source of income, less likely to have a community services card, and lived in less deprived areas but interestingly were just as likely to report inadequate or barely adequate financial resources. Those patients on home dialysis were also more likely to be male, be of NZ European descent and to live further from the dialysis centre. The pre-dialysis population was similar to the peritoneal dialysis population with similar apparent socioeconomic advantage over the in-centre dialysis population. While the population undertaking in-centre haemodialysis was different than the home or pre-dialysis population, dialysis vintage was associated with no significant demographic differences.

Despite the differences in socioeconomic status, multiple measures of health and disability status did not vary by being on dialysis, by dialysis modality, dialysis location of time on renal replacement therapy. This would imply that factors outside of socioeconomic status have more influence on these characteristics.

Strengths of this study are that we achieved good representation of the NZ dialysis population over the age of 65. Importantly, participation of Māori and Pacific people were at rates higher, or equivalent to, rates of renal replacement therapy in these ethnic groups. Comorbidity data was individually collected from patient hospital records. Multiple measures of socioeconomic status were collected either directly from the participant or from national census data. Prioritised ethnicity data was collected directly from the participant which has been shown to be more reliable than electronic health records [6]. Multiple measures of health status, HRQoL, disability and personal well-being were recorded by trained interviewers.

In NZ dialysis is entirely provided within the publicly- funded health system and therefore decisions related to acceptance onto dialysis, the modality of dialysis, and subsequent participation in this study, is not subject to participant insurance status or other financial factors.

While those who participated were representative of the NZ wide dialysis population of a similar age, females, Māori and Pacific people and those not on dialysis were less likely to participate. In particular, a relatively low number of Māori and Pacific who were either undertaking peritoneal dialysis or who were pre-dialysis participated. This may limit the generalisability of findings for these populations, though as a prospective cohort study, participation bias has little concern when looking at risk factors for outcomes of interest that have not yet occurred.

Our future analyses will concentrate on characterizing the evolution of patient-related over time. Our study has an accelerated longitudinal design, which is similar to that used in the Dialysis Outcomes and Practice Patterns Study, where analyses are also made on a mix of incident and prevalent dialysis patients [3]. The difference is that in the DOPPS, most analyses are performed using time-to-event models for outcomes such as mortality. As such, patients are “lined up” at their time of study entry, controlling for vintage (on a continuous scale) by including it in a model. In our study, most analyses will be observations of patient-related outcomes at various follow-up points post-dialysis inception. As such, patients will be “lined up” by vintage. For these sorts of outcomes, there is no difference between the accelerated longitudinal design used in our study versus that which using an incident or inception cohort design – in our study, prevalent patients recruited at year 2 post-dialysis inception followed for two years with have the same cross-sectional characteristics at each landmark as incident patients recruited at dialysis inception followed for four years. In both cases, there is no issue with survival bias; for instance, one can only compare HRQoL at a landmark in patients who have survived to that point, and no other analytical framework makes clinical sense. Table 2 demonstrates that patients can be pooled without any sub-cohort effect related the sampling frame.

In the case that we analyse follow-up data using time-to-event models, for instance to assess mortality, we will use similar methods to the DOPPS [3] with left truncation (at time of study entry) in to account for the potential bias of missing patients who did not make it to the point of study entry. In such analyses, survival time is conditional on having already survived from the point of risk (dialysis inception) to first coming under observation (study entry). In New Zealand, less than 2% of the dialysis population aged 65 or older receive a renal transplant (4), and the competing risk of transplant will not be a factor in analysing outcomes in this study.

## Conclusions

In summary, we report the baseline characteristics of participants enrolled prospectively into a longitudinal cohort observation study examining HRQoL factors with clinical characteristics on dialysis outcomes in a group of New Zealand aged 65 years or older who are either on dialysis or have been educated about dialysis [2]. In combination with our protocol paper [2], this paper allows readers a clear understanding of the participants enrolled in the study, in order to compare with their own clinical practice. Subsequent publications are planned, analysing the prospective longitudinal data to identify key factors that determine both outcome and quality of life for individuals of this age group.

## Abbreviations

ANOVA: Analysis of variance
ANZDATA: Australian and New Zealand Dialysis and Transplantation Registry
CKD: Chronic kidney disease
CVD: Cardiovascular disease
DHB: District Health Board
DOPPS: Dialysis outcomes and practice patterns study
DOS65: Dialysis outcomes in those aged 65 years or older study
eGFR: estimated glomerular filtration rate
EQ-5D-3 L: EuroQual questionnaire
ESKD: End stage kidney disease
FACIT: Functional assessment of chronic illness therapy
HRQoL: Health related quality of life
IQR: Interquartile range
KDQoL − 36: Kidney disease quality of life 36 questionnaire
NZ: New Zealand
NZDep: New Zealand deprivation index
SONG: Standardised outcomes in Nephrology
